# Effect of Fentanyl as an Adjuvant to Brachial Plexus Block for Upper Extremity Surgeries: A Systematic Review and Meta-Analysis of RCTs

**DOI:** 10.1155/2022/8704569

**Published:** 2022-03-19

**Authors:** Liangsong Song, Shulian Tan, Qingmin Chen, He Li

**Affiliations:** ^1^Department of Hand and Foot Surgery, The First Hospital, Jilin University, Changchun, China; ^2^Institute of Immunology, The First Hospital, Jilin University, Changchun, China; ^3^Department of Hepatopancreatobiliary Surgery, The First Hospital, Jilin University, Changchun, China; ^4^Department of Pain Medicine, The First Hospital, Jilin University, Changchun, China

## Abstract

**Objective:**

To assess if the addition of fentanyl to brachial plexus block has an impact on anesthetic outcomes and complication rates in patients undergoing upper extremity surgeries.

**Methods:**

We explore the PubMed, Embase, ScienceDirect, CENTRAL, and Google Scholar databases for all randomized controlled trials (RCTs) comparing adjuvant fentanyl with placebo/no drug for patients undergoing upper extremity surgery under brachial plexus block. Outcomes assessed were onset, duration of sensory and motor anesthesia, complications, and postoperative analgesia scores. Meta-analysis was conducted utilizing a random-effects model. The risk of bias was assessed using the Cochrane Collaboration's risk of bias assessment tool 2. Certainty of evidence was assessed using GRADE. Subgroup analysis was conducted depending upon the approach of brachial plexus block and type of local anesthetic.

**Results:**

Twelve RCTs with 660 patients were included. Addition of fentanyl had no effect on onset of sensory anesthesia (11 studies; MD: 0.48; 95% CI: −1.81, 0.85; *I*^2^ = 96%; *p*=0.48) but significantly shortened onset of motor anesthesia (8 studies; MD: −2.36; 95% CI: −3.99, −0.74; *I*^2^ = 96%; *p*=0.48). Duration of sensory anesthesia (9 studies; MD: 82.81; 95% CI: 41.81, 123.81; *I*^2^ = 99%; *p* < 0.0001) and motor anesthesia (7 studies; MD: 93.41; 95% CI: 42.35, 144.46; *I*^2^ = 99%; *p*=0.0003) was significantly increased with addition of fentanyl. The certainty of evidence-based on GRADE was deemed to be moderate for both onset and duration of anesthesia. The incidence of overall complications (nausea/vomiting and pruritis) was significantly higher in the fentanyl group (7 studies; OR: 2.14; 95% CI: 1.04, 4.40; *I*^2^ = 8%; *p*=0.04) but with low certainty of evidence.

**Conclusions:**

Adjuvant fentanyl with brachial plexus block improves the onset of motor anesthesia but not sensory anesthesia. The duration of both sensory and motor anesthesia is significantly prolonged with fentanyl by around 83–93 minutes. However, clinicians should be aware that complications such as nausea/vomiting and pruritis are increased twofold with the addition of the drug. Current evidence is limited risk of bias in the RCTs and high heterogeneity in the meta-analyses.

## 1. Introduction

Regional anesthesia techniques offer several advantages over general anesthesia by limiting the area anesthetized, thereby increasing patients' satisfaction, reducing the length of hospital stay, and lowering overall healthcare costs [1]. One such technique is the brachial plexus block that has become the primary anesthetic modality for most upper extremity surgeries [2]. The block may be administered via the supraclavicular, infraclavicular, axillary, or interscalene approach with each technique having its advantages and disadvantages [3]. The addition of ultrasound has improved the success of the technique by allowing the operator to visualize the needle position and administer the block in patients with anatomical variation [4]. However, a limitation of plain local anesthetic (LA) brachial plexus block, or for that matter any peripheral nerve block, is the short duration of anesthesia even with long-acting agents. The effect of anesthesia is known to wear off in some hours, thereby exposing the patient to the pain of surgical trauma [5]. One method to prolong the anesthesia duration is to increase the dose of the LA but with a corresponding increase in cardiovascular and central nervous system toxicity [6]. Another more acceptable method is to use drug adjuvants to prolong the duration of anesthesia without concomitantly increasing the risk of complications. Over the years, a myriad of adjuvant drugs has been used to complement peripheral nerve blocks including the brachial plexus block but with conflicting evidence [7, 8].

Opioids have been a popular class of drugs for pain control in surgical and non-surgical patients for several decades [9]. Systemically administered opioids act mainly through the activation of opioid receptors located in the central nervous system. However, it is now well known that these receptors are present in peripheral neurons as well [10]. Targeting the peripheral receptors can avoid debilitating centrally mediated adverse events such as respiratory depression, altered consciousness, and addiction leading to improved analgesia [11]. Indeed, owing to the high potency of these drugs, several different opioids have also been used as adjuvants to LA for various peripheral nerve blocks [12, 13].

In the case of brachial plexus blocks, some of the commonly used opioid adjuvants include morphine, meperidine, buprenorphine, nalbuphine, and fentanyl [8, 12]. As compared to the naturally occurring parent opioid morphine, fentanyl is a synthetic opioid agonist that has high potency and faster onset of action [14]. When used intrathecally, fentanyl produces better intraoperative and postoperative analgesia without any hemodynamic instability. The better safety profile of the drug has made it a popular choice as compared to other opioids [15]. Over the past two decades, several studies have evaluated the effects of fentanyl added to brachial plexus block but with small sample sizes and variable results. Currently, it is unclear how exactly fentanyl impacts the outcomes of this nerve block. Furthermore, to the best of our knowledge, no systematic review has evaluated evidence on the use of adjuvant fentanyl for brachial plexus block. Given this deficiency in literature, we conducted this systematic review and meta-analysis of randomized controlled trials (RCTs) to clarify the influence of adjuvant fentanyl on anesthetic outcomes of brachial plexus block.

## 2. Material and Methods

This review conforms to the guidelines of the PRISMA statement (Preferred Reporting Items for Systematic Reviews and Meta-Analyses) [16] and the Cochrane Handbook for Systematic Reviews of Intervention [17]. The review aimed to answer the following clinical query: does the addition of fentanyl to brachial plexus block impact anesthetic outcomes and complication rates in patients undergoing upper extremity surgeries?

### 2.1. Literature Search

We examined the databases of PubMed, Embase, ScienceDirect, CENTRAL, and Google Scholar for studies eligible for the review. The search was conducted electronically beginning from 1 January 2000 to 15 March 2021. These limits were common for all databases. We looked for any ongoing trial on clinicaltrials.gov; however, only article preprints that were not peer-reviewed were excluded. A mix of relevant keywords, namely, “fentanyl,” “opioids,” “brachial plexus block,” “axillary block,” “supraclavicular block,” and “interscalene block,” were used for the database searches. Exact search queries are presented in Supplementary Table 1. To ensure thoroughness and bias-free results, two reviewers were independently involved in the database searches. The initial results were then deduplicated electronically using EndNote software X7 (Clarivate Analytics, USA). The first batch of search results were screened only by article titles and abstracts to look for studies relevant to the review. Once identified, we downloaded the full texts of appropriate studies and analyzed them against our inclusion criteria. This was done by the two reviewers separately. Differences if any were resolved by discussion. In the last step, we examined the bibliography of included studies to look if any other studies were missed.

### 2.2. Inclusion Criteria

A PICOS (Population, Intervention, Comparison, Outcome, and Study design) based inclusion criteria was formulated as follows:

 
*Population*: patients undergoing any kind of upper extremity surgery under brachial plexus block
 
*Intervention*: any dose of fentanyl as an additive to the nerve block
 
*Comparison*: placebo or no drug
 
*Outcomes*: onset or duration of anesthesia, postoperative pain scores or analgesic consumption, and complications
 
*Study design*: RCTs

Exclusion criteria were as follows: (1) intravenous or intramuscular administration of fentanyl, (2) uncontrolled studies comparing fentanyl with any other active drug, (3) not reporting relevant outcomes, (4) non-RCTs, retrospective studies, and (5) non-English language studies.

### 2.3. Data Extraction and Risk of Bias Assessment

A data extraction sheet was prepared at the beginning of the review, and this was used by two reviewers to extract relevant data. Details of the study including author(s), study location, approach of brachial plexus block, number of patients, age and gender, type and dose of LA used, fentanyl dose, any other anesthetic or sedative agents used, postoperative analgesia, and study outcomes were extracted. The study authors were not contacted for any missing data. Data extraction was carried out manually, and no automation tools were used. The primary outcomes of interest were the onset and duration of sensory and motor anesthesia. Secondary outcomes of interest were postoperative pain scores or analgesic requirement and incidence of complications related to the addition of fentanyl. We did not define the outcomes for the review, and the definition of the outcomes as per the included studies was accepted. Data were extracted as directly reported by the studies, and no assumptions were made. If the data could not be analyzed quantitatively, only a descriptive analysis was carried out.

Since only RCTs were eligible for the review, two reviewers used the Cochrane Collaboration's risk of bias assessment tool 2 to assess the quality of studies [17]. The tool has five domains for quality evaluation, namely, randomization process, deviation from intended intervention, missing outcome data, measurement of outcomes, and selection of reported results. Differences between the reviewers for data extraction or quality assessment were resolved by consensus. The Grading of Recommendations Assessment, Development, and Evaluation (GRADE) tool available on the GRADEpro GDT software (GRADEpro Guideline Development Tool; McMaster University, 2020 (developed by Evidence Prime, Inc.)) was used for evaluating the certainty of evidence.

### 2.4. Statistical Analysis

All quantitative analyses were conducted using “Review Manager” (RevMan, version 5.3; Nordic Cochrane Centre (Cochrane Collaboration), Copenhagen, Denmark; 2014). All outcome data from the studies were tabulated in order to assess the studies eligible for meta-analysis of each outcome. Data on onset and duration of anesthesia were summarized using mean difference (MD) with 95% confidence intervals (CI). Complications were summarized using odds ratios (OR) with 95% CI. All continuous data (duration and onset time) were expressed in minutes. In some studies, outcome data were reported only as figures. In such cases, Engauge Digitizer Version 12.1 was used to get numerical data. When continuous variables were reported as median, range, and interquartile range by the included studies, it was transformed into mean and standard deviation (SD) by methods of Wan et al. [18]. We generated forest plots using the meta-analysis software to display the results of each outcome. All analyses were carried out using a random-effects model.

To assess for interstudy heterogeneity, subgroup analyses were carried out based on the approach of brachial plexus block and type of LA. A sensitivity analysis was carried out for the outcomes of onset and duration of anesthesia by excluding one study at a time in the meta-analysis software itself to look for any change in the significance of the results. The *I*^2^ statistic was used to assess heterogeneity amongst the studies. *I*^2^ scores of 25–50% denoted low, while values of 50–75% and >75% denoted medium and substantial heterogeneity, respectively. Funnel plots were used to assess publication bias.

## 3. Results

### 3.1. Details of Included Studies

**Figure 1:**
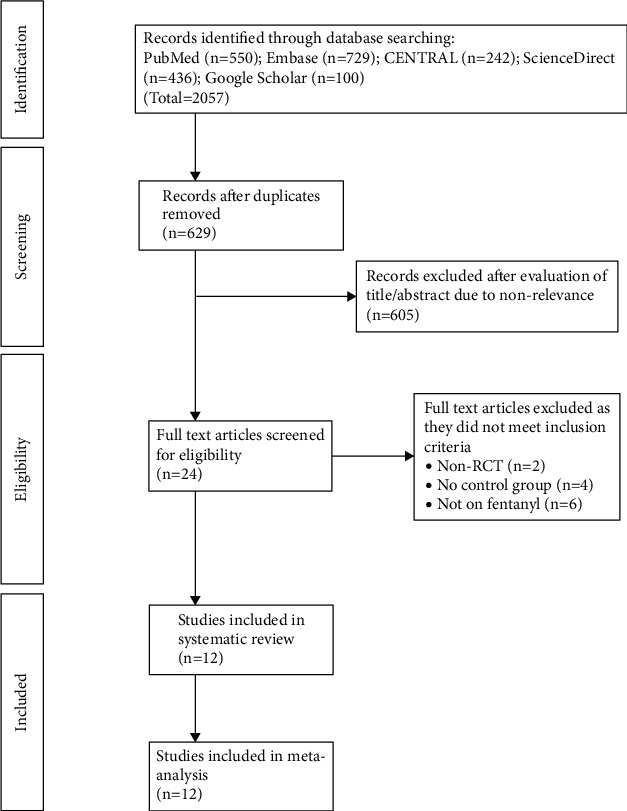
Study flow chart.

We retrieved 2,057 articles after the literature search (Figure 1). These were deduplicated, and a total of 629 unique records were examined by their titles and abstracts. A total of 605 articles were excluded as they were not relevant to the review. Twenty-four articles were selected for full-text review of which 12 RCTs fulfilled the inclusion criteria [19–30]. A total of 12 studies were excluded with reasons (2 studies were not RCTs, 4 studies had no control group, and 6 studies were not on fentanyl). Baseline details of the studies are presented in Table 1. The included studies were published between 2000 and 2018 and were conducted in India, Iran, Egypt, Italy, Turkey, and Japan. Supraclavicular and axillary approaches were most commonly used, while only 1 study used the interscalene approach. There was wide variation in the type and dosage of LA used by the RCTs, which included lidocaine, bupivacaine, levobupivacaine, and ropivacaine. The most common doses of fentanyl used by the trials were 1 µg/kg or a fixed dose of 100 µg. None of the trials used general anesthesia along with the nerve blocks. A few trials reported the use of midazolam to sedate the patients before the nerve blocks, while only 1 trial [28] reported the use of diazepam during the surgical procedure.

### 3.2. Risk of Bias

**Figure 2:**
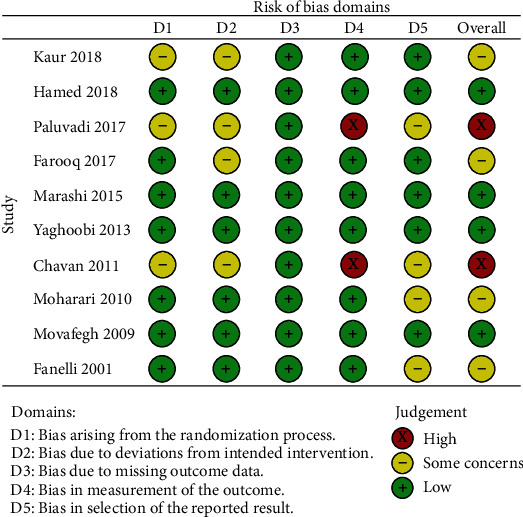
Risk of bias plot for the included RCTs.

Details of risk of bias in the included RCTs as per the review author's judgment are presented in Table 2. Four trials each had “some concerns” related to the randomization process, deviations from the intended intervention, and selection of reported results. There was no risk of bias due to missing outcomes. Two studies had a high risk of bias for the measurement of outcomes. Overall, two trials were marked with a high risk of bias, while five trials had “some concerns” (Figure 2).

### 3.3. Onset of Anesthesia

**Figure 3:**
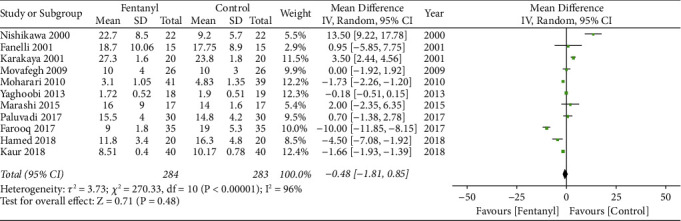
Meta-analysis of onset of sensory anesthesia between fentanyl and control groups.

**Figure 4:**
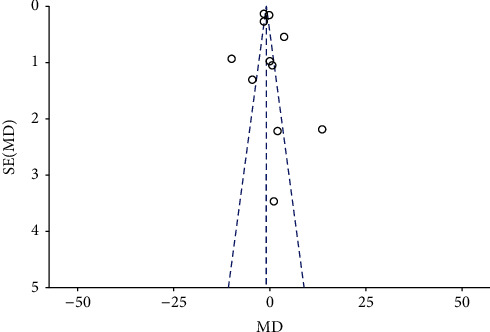
Funnel plot for meta-analysis of onset of sensory anesthesia.

A total of 11 RCTs with 284 patients in the fentanyl group and 283 patients in the control group reported data on the onset of sensory anesthesia. Pooled analysis denoted no statistically significant difference between fentanyl and control groups (MD: 0.48; 95% CI: −1.81, 0.85; *I*^2^ = 96%; *p*=0.48; Figure 3). We did not detect any publication bias (Figure 4). During sensitivity analysis, on the exclusion of the study of Farooq et al. [22], the results indicated significantly reduced time for sensory onset with adjuvant fentanyl (MD: −1.28; 95% CI: −2.56, −0.01; *I*^2^ = 96%; *p*=0.05). On subgroup analysis based on the approach of brachial plexus block, the onset of sensory anesthesia was significantly longer in the fentanyl group for the axillary approach but insignificant for the supraclavicular approach (Table 3). On the other hand, there was no difference based on the type of LA agent used.

**Figure 5:**
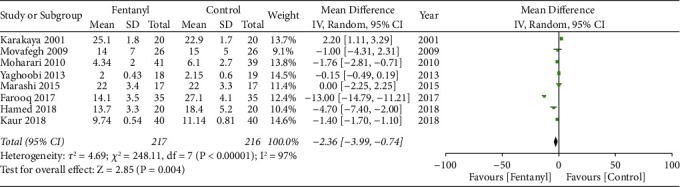
Meta-analysis of onset of motor anesthesia between fentanyl and control groups.

**Figure 6:**
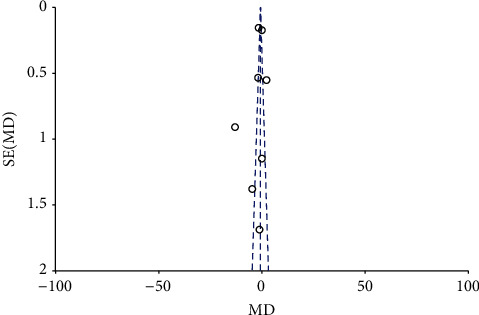
Funnel plot for meta-analysis of onset of motor anesthesia.

On the pooling of data from 8 RCTs with 217 patients in the fentanyl group and 216 patients in the control group, we found a statistically significant faster onset of motor anesthesia in the fentanyl group (MD: −2.36; 95% CI: −3.99, −0.74; *I*^2^ = 96%; *p*=0.48; Figure 5). However, the effect size was no longer significant on subgroup analysis based on the block approach and type of LA (Table 3). The funnel plot did not indicate any publication bias (Figure 6). During sensitivity analysis, exclusion of the studies of Farooq et al. [22] (MD: −0.71; 95% CI:−1.73, 0.31; *I*^2^ = 92%; *p*=0.17) and Kaur et al. [19] (MD: −2.60; 95% CI:−5.43, 0.24; *I*^2^ = 97%; *p*=0.07) demonstrated no difference in motor onset between the two groups. The GRADE assessment indicated the certainty of evidence to be moderate for both sensory and the motor onset of anesthesia (Supplementary Table 2).

### 3.4. Duration of Anesthesia

**Figure 7:**
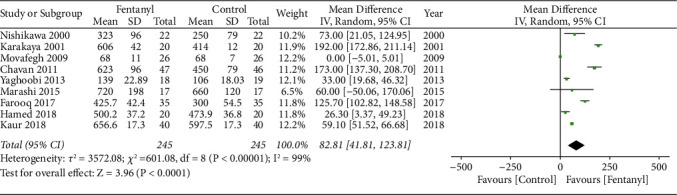
Meta-analysis of the duration of sensory anesthesia between fentanyl and control groups.

**Figure 8:**
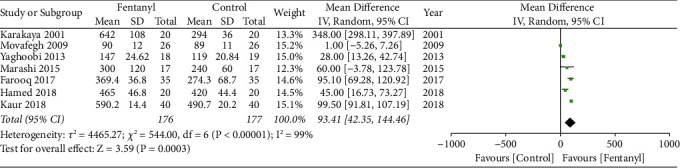
Meta-analysis of the duration of motor anesthesia between fentanyl and control groups.

**Figure 9:**
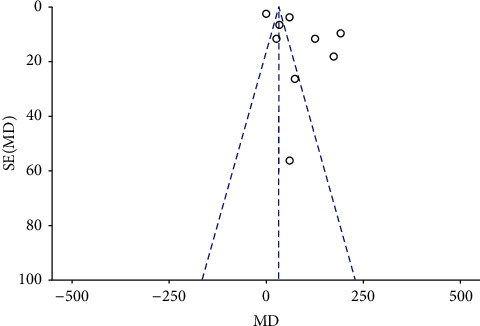
Funnel plot for meta-analysis of the duration of sensory anesthesia.

**Figure 10:**
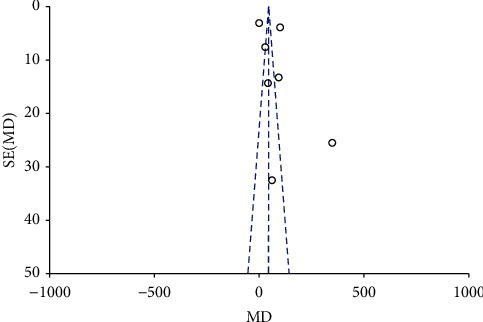
Funnel plot for meta-analysis of the duration of motor anesthesia.

We found that the addition of fentanyl to brachial plexus block significantly increased the duration of sensory anesthesia (MD: 82.81; 95% CI: 41.81, 123.81; *I*^2^ = 99%; *p* < 0.0001; Figure 7) as well as motor anesthesia (MD: 93.41; 95% CI: 42.35, 144.46; *I*^2^ = 99%; *p*=0.0003; Figure 8). There were some evidence of publication bias for both outcomes (Figures 9 and 10). The results did not change on sensitivity analysis. On subgroup analysis, the duration of sensory and motor anesthesia was prolonged for both supraclavicular and axillary approaches. However, the difference was insignificant for studies using lidocaine as compared to levobupivacaine/bupivacaine for both outcomes (Table 3). The certainty of evidence based on GRADE was deemed to be moderate for both sensory and motor duration of anesthesia (Supplementary Table 2).

### 3.5. Complications

**Figure 11:**
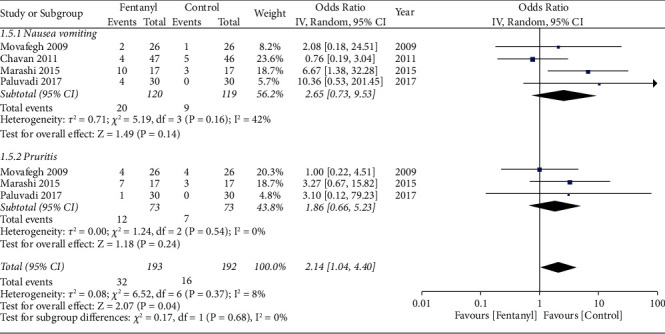
Meta-analysis of complications between fentanyl and control groups.

Data on complications was reported by very limited studies. Nausea/vomiting and pruritis were the most common complications reported. The incidence of overall complications was significantly higher in the fentanyl group (OR: 2.14; 95% CI: 1.04, 4.40; *I*^2^ = 8%; *p*=0.04; Figure 11). However, subgroup analysis indicated a non-significant but tendency of higher odds of nausea/vomiting (OR: 2.65; 95% CI: 0.73, 9.53; *I*^2^ = 42%; *p*=0.14) and pruritis (OR: 1.86 95% CI: 0.66, 5.23 *I*^2^ = 0% *p*=0.24) in the fentanyl group.). The GRADE assessment indicated the certainty of evidence to be low (Supplementary Table 2).

### 3.6. Postoperative Analgesia

Due to heterogeneous and limited reporting, we could not quantitatively analyze the effect of fentanyl on postoperative analgesia. Outcomes of individual studies are presented in Table 4. On descriptive analysis, the impact of fentanyl was not found to be consistent with some studies reporting better analgesic effects in the fentanyl group while others reporting no difference.

## 4. Discussion

Our first systematic review and meta-analysis analyzing the impact of adjuvant fentanyl to a brachial plexus block indicate that fentanyl results in a significantly faster onset of motor anesthesia but not sensory anesthesia. The duration of sensory anesthesia is significantly increased by 83 minutes ranging from 42 to 124 minutes, while the duration of motor anesthesia is increased by 93 minutes ranging from 42 to 145 minutes. Limited data suggest that the incidence of overall complications consisting mainly of nausea/vomiting and pruritis is increased by twofold in patients receiving fentanyl. The overall certainty of evidence ranged from moderate to low.

Several surgeries of the upper extremity are now performed under day-care anesthesia with the aid of brachial plexus block [2]. However, despite the use of longer-acting LA, the anesthetic effect frequently wears off after several hours leading to postoperative pain. Therefore, improving the duration and onset of brachial plexus block has been an important area of research in the last decade [5]. Ideally, the adjuvant drug should significantly prolong the duration of anesthesia, shorten its onset, and increase the time to first analgesic request without any significantly increased risk of complication. Of the several drugs available for an anesthesiologist, fentanyl has been used as an adjuvant to several different regional anesthesia techniques over many years [31–33]. Sindjelic et al. [31] have reported that the use of adjuvant fentanyl with cervical plexus block significantly reduces propofol consumption along with postoperative analgesic demand. Terkawi et al. [33] in a meta-analysis of various techniques to improve the quality of thoracic paravertebral nerve blocks for breast surgery patients found that only adjuvant fentanyl and the use of multilevel blocks were associated with improved pain control. In another study, Abbi et al. [34] have shown that combined fentanyl and bupivacaine significantly shortened the onset and prolonged the duration of spinal anesthesia in patients undergoing lower limb surgeries as compared to bupivacaine alone. However, the effect of perineural fentanyl has not been consistent across literature with trials also indicating that the opioid may not have any prominent analgesic effect with peripheral nerve blocks [35].

Such inconsistency in the effect of adjuvant opioids may be attributed partly to the small sample size of many RCTs in the literature. As seen in our review, almost half of the studies included <25 patients per arm. In this context, a meta-analysis becomes important to present high-quality evidence to practicing clinicians. The results of our review indicate that adjuvant fentanyl significantly increases the duration of both motor and sensory anesthesia. This effect was consistently seen across the majority of studies in the review despite the high heterogeneity in the meta-analysis. The beneficial effects of fentanyl with peripheral nerve blocks have been attributed to its direct action on peripheral opioid receptors [5]. The drug may also penetrate the nerve membrane and act directly on the dorsal horn owing to the bidirectional axonal transport of opioid binding proteins leading to a significant increase in the duration of nerve block [30]. We also noted that the onset of motor anesthesia was significantly reduced, but there was no effect on the onset of sensory anesthesia. While such a result is difficult to explain, we noticed that delayed onset of sensory anesthesia was seen with fentanyl in earlier studies, while faster onset was demonstrated in more recent studies. It may be speculated that the changes in drug formulations and pH of the solution may have contributed to this difference.

Though our review focused solely on a single opioid, the effect size of fentanyl needs to be viewed concerning other additives that have been used with brachial plexus block. Choi et al. [36] in a meta-analysis of nine RCTs have demonstrated that the addition of dexamethasone to brachial plexus block significantly increases the duration of sensory and motor anesthesia by 410 and 438 minutes, respectively. Another meta-analysis of 18 RCTs has indicated that dexmedetomidine also has a similar effect by prolonging the duration of sensory anesthesia by 261 minutes and motor anesthesia by 201 minutes [37]. On the other hand, tramadol, which is another popular opioid used as an adjuvant by multiple studies, increases the length of sensory and motor blockade by 62 and 66 minutes, respectively [38]. Thus, with an effect size of 83 minutes for the duration of sensory and 93 minutes for the duration of motor block, by indirect comparison, fentanyl ranks just above tramadol but much lower as compared to dexamethasone and dexmedetomidine.

An important point of consideration while interpreting our results is the high heterogeneity noted in all our meta-analyses. This can be attributed to the different types, concentrations, and doses of LA used; to the difference in the block approach; and also to the minor variation in the fentanyl dosage in the included trials. Furthermore, it may also have been influenced by the anesthetic protocol and the difference in the definition of outcomes as the heterogeneity persisted even with a subgroup analysis. Comparing the different types of LA solutions, it is well-known that lidocaine has a shorter duration of action as compared to bupivacaine or ropivacaine [39]. Amongst the two long-acting agents, ropivacaine has a significantly shorter onset of action, but bupivacaine has a longer duration of anesthesia for brachial plexus blocks [40]. Due to a limited number of studies, we were unable to carry out a subgroup analysis for all outcomes for ropivacaine, but our results indicated that the addition of fentanyl improves the duration of anesthesia only with bupivacaine/levobupivacaine but not with lidocaine. Contrastingly, the use of dexamethasone improves the duration of the block with both intermediate and long-acting LA agents [36]. We believe the small effect size of fentanyl as compared to other agents may have contributed to this result. In the other subgroup analysis, there was no difference in the duration of nerve blocks administered via axillary or supraclavicular approach. This is in agreement with studies indicating that the approach of brachial plexus block does not affect anesthetic outcomes [41, 42].

The use of opioids is known to cause several minor and major side effects [43]. While none of the included studies reported major adverse outcomes such as respiratory depression, hypotension, and so on with the use of fentanyl, the incidence of complications such as nausea/vomiting and pruritis was increased twofold with the addition of fentanyl to brachial plexus block. Individual analysis of these complications was non-significant probably due to the limited data available and the small sample size of the studies.

There are some limitations to our review. Foremost, as mentioned above, the results must be interpreted with caution owing to the high heterogeneity of our analysis. Despite adding subgroup analysis to the review, we were unable to eliminate it. Secondly, the number of included studies was not very high. Due to the unavailability of data, not all studies could be included in all the meta-analyses. Thirdly, all the included RCTs were not of high-quality based on the recently developed risk of bias-2 tool. Only 5 of the 12 studies were judged to have a low risk of overall bias. Lastly, owing to limited data, we were unable to quantitatively analyze postoperative pain scores and analgesic requirements of the two study groups. One reason for the limited number of RCTs analyzing the effect of fentanyl could be the myriad of drugs available for use as an adjuvant to brachial plexus block. These include various non-opioids such as dexamethasone, dexmedetomidine, and clonidine that are more commonly used in clinical practice. However, to date, there has been limited literature on the comparative efficacy of fentanyl vis-à-vis other adjuvants for brachial plexus blocks. Only future research comparing the efficacy of different drugs with fentanyl in large RCTs can provide concrete evidence on this topic.

The clinical relevance of the current meta-analysis is derived from the fact that despite fentanyl being used as an adjuvant to brachial plexus block, to date, there has been no systematic effort to establish its efficacy. Our review is the first to pool data from all RCTs conducted to date to assess the impact of adjuvant fentanyl on the onset and duration of brachial plexus blocks. A detailed systematic search was performed to include only RCTs, thereby providing level-1 evidence. Appropriate subgroup analysis and GRADE assessment were also performed. We believe our meta-analysis shall aid clinicians to make informed decisions on the use of fentanyl with brachial plexus blocks.

## 5. Conclusions

The addition of fentanyl to LA in brachial plexus block improves the onset of motor anesthesia but not sensory anesthesia. The duration of both sensory and motor anesthesia is significantly prolonged with fentanyl by around 83–93 minutes. However, clinicians should be aware that complications such as nausea/vomiting and pruritis are increased twofold with the addition of the drug.

## Figures and Tables

**Table 1:** Details of included studies.

Study	Location	Approach of brachial plexus block	Local anesthetic used	Dose of fentanyl	Other anesthetic/sedative used	Sample size		Mean age (years)		Male gender (%)		Postoperative analgesic
						Study	Control	Study	Control	Study	Control	
Kaur et al. [19]	India	Supraclavicular	25 ml of 0.5% levobupivacaine	1 *µ*g/kg	NR	40	40	NR	NR	NR	NR	Inj PCM IV 1 g if VAS > 3
Hamed et al. [20]	Egypt	Supraclavicular	Up to 40 ml of 0.5% bupivacaine	1 *µ*g/kg	NR	20	20	32.35 ± 9.2	30.2 ± 9.6	70	75	Inj Diclofenac IM 1 mg/kg if VA S> 4
Paluvadi et al. [21]	India	Supraclavicular	30 ml of 1.5% lidocaine	1 *µ*g/kg	NR	30	30	42.6 ± 11.8	38.16 ± 12.2	76.6	76.6	NR
Farooq et al. [22]	India	Supraclavicular	3 mg/kg of 0.75% ropivacaine	1 *µ*g/kg	IV Midazolam 2 mg (before block)	35	35	38.3 ± 11.2	38.3 ± 11.2	54.2	54.2	NR
Marashi et al. [23]	Egypt	Supraclavicular	30 ml of 0.5% bupivacaine	100 *µ*g	IV Midazolam 1 mg (before block)	17	17	32.2 ± 9	32.7 ± 10	52.9	58.8	Morphine PCA
Yaghoobi et al. [24]	Iran	Axillary	40 ml of 1% lidocaine	100 *µ*g	IV Midazolam 1 mg (before block)	18	19	31.28 ± 6.05	30.01 ± 5.47	77.7	73.7	Pethidine 25 mg
Chavan et al. [25]	India	Supraclavicular	20 ml of 0.5% bupivacaine plus 10 ml of 2% lignocaine	50 *µ*g	NR	47	46	46 ± 16	48 ± 17	51	52.1	NR
Moharari et al. [26]	Iran	Interscalene	30 ml of 1.5% lidocaine	75 *µ*g	IV Diazepam 10 mg (during surgery)	41	39	44.89 ± 11.38	41.46 ± 10.93	53.6	56.4	NR
Movafegh et al. [27]	Iran	Axillary	34 ml of 1.5% lidocaine	100 *µ*g	IV Midazolam 1 mg (before block)	26	26	34.1 ± 10.7	33.3 ± 7.6	57.7	53.8	NR
Fanelli [29]	Italy	Axillary	20 ml of 0.75% ropivacaine	1 *µ*g/ml	IV Midazolam 0.05 mg/kg (before block)	15	15	56 ± NR	53 ± NR	53.3	60	Inj Ketoprofen IV 100 mg
Karakaya et al. [28]	Turkey	Axillary	40 ml of 0.25% bupivacaine	2.5 *µ*g/ml	None	20	20	38.7 ± 2.5	41.5 ± 3.8	50	55	NR
Nishikawa et al. [30]	Japan	Axillary	40 ml of 1.5% lidocaine	100 *µ*g	IV Midazolam 2.5 mg (before block)	22	22	56 ± 16	58 ± 17	54.5	50	NR

Note: IV, intravenous; IM, intramuscular; PCM, paracetamol; PCA, patient-controlled analgesia; NR, not reported; and VAS, visual analog scale.

**Table 2:** Risk of bias in included studies.

Study	Randomization process	Deviation from intended intervention	Missing outcome data	Measurement of outcomes	Selection of reported result	Overall risk of bias
Kaur et al. [19]	Some concerns	Some concerns	Low risk	Low risk	Low risk	Some concerns
Hamed et al. [20]	Low risk	Low risk	Low risk	Low risk	Low risk	Low risk
Paluvadi et al. [23]	Some concerns	Some concerns	Low risk	High risk	Some concerns	High risk
Farooq et al. [24]	Low risk	Some concerns	Low risk	Low risk	Low risk	Some concerns
Marashi et al. [25]	Low risk	Low risk	Low risk	Low risk	Low risk	Low risk
Yaghoobi et al. [26]	Low risk	Low risk	Low risk	Low risk	Low risk	Low risk
Chavan et al. [27]	Some concerns	Some concerns	Low risk	High risk	Some concerns	High risk
Moharari et al. [28]	Low risk	Low risk	Low risk	Low risk	Some concerns	Some concerns
Movafegh et al. [29]	Low risk	Low risk	Low risk	Low risk	Low risk	Low risk
Fanelli [21]	Low risk	Low risk	Low risk	Low risk	Some concerns	Some concerns
Karakaya et al. [30]	Some concerns	Low risk	Low risk	Low risk	Low risk	Some concerns
Nishikawa et al. [22]	Low risk	Low risk	Low risk	Low risk	Low risk	Low risk

**Table 3:** Subgroup analysis of the study outcomes.

Variable	Category	Number of studies	Effect size (mean difference)
*Sensory onset*			
Block approach	Supraclavicular Axillary	5 5	−2.86 (95% CI −6.51, 0.79); *I*^2^ = 95%; *p*=0.123.14 (95% CI 0.20, 6.06); *I*^2^ = 95%; *p*=0.04
Local anesthetic	Lidocaine Levobupivacaine/bupivacaine Ropivacaine	5 4 2	0.95 (95% CI −0.73, 2.62); *I*^2^ = 94%; *p*=0.27 −0.21 (95% CI −3.74, 3.32); *I*^2^ = 97%; *p*=0.91 −5.03 (95% CI −15.72, 5.65); *I*^2^ = 89%; *p*=0.36

*Motor onset*			
Block approach	Supraclavicular Axillary	4 3	−4.77 (95% CI −10.55, 1.00); *I*^2^ = 98%; *p*=0.11 0.58 (95% CI −1.35, 2.52); *I*^2^ = 88%; *p*=0.55
Local anesthetic	Lidocaine Levobupivacaine/bupivacaine	3 4	−0.88 (95% CI −2.21, 0.46); *I*^2^ = 76%; *p*=0.20 −0.80 (95% CI −3.20, 1.61); *I*^2^ = 94%; *p*=0.52

*Sensory duration*			
Block approach	Supraclavicular Axillary	5 5	90.66 (95% CI 44.50, 136.83); *I*^2^ = 95%; *p* = 0.001 93.70 (95% CI 18.87, 168.53); *I*^2^ = 99%; *p* = 0.01
Local anesthetic	Lidocaine Levobupivacaine/bupivacaine	3 4	27.33 (95% CI −4.49, 58.96); *I*^2^ = 93%; *p*=0.001 86.65 (95% CI 8.49, 164.82); *I*^2^ = 98%; *p*=0.03

*Motor duration*			
Block approach	Supraclavicular Axillary	4 3	95.26 (95% CI 88.17, 102.34); *I*^2^ = 79%; *p* < 0.00001 9.63 (95% CI 3.91, 15.36); *I*^2^ = 99%; *p*=0.001
Local anesthetic	Lidocaine Levobupivacaine/bupivacaine	2 4	5.12 (95% CI −0.64, 10.88); *I*^2^ = 91%; *p*=0.08 100.67 (95% CI 93.38, 107.96); *I*^2^ = 97%; *p* < 0.00001

**Table 4:** Postoperative analgesic outcomes.

Study	Outcome	Result
Kaur et al. [19]	Number of rescue analgesic injection	Significantly lower in the fentanyl group (*p* < 0.05)
Farooq et al. [24]	Time for first rescue analgesic	Significantly higher in the fentanyl group (*p* < 0.05)
Marashi et al. [25]	Postoperative pain scores	No statistically significant difference between the study groups
Yaghoobi et al. [26]	Total analgesic consumption Pain score at the return of sensation	Significantly lower in the fentanyl group (*p* < 0.05) Significantly lower in the fentanyl group (*p* < 0.05)
Moharari et al. [28]	Time for first rescue analgesic	Significantly higher in the fentanyl group (*p* < 0.05)
Fanelli [21]	Time for first rescue analgesic Pain score at first request for analgesic Total analgesic consumption	No statistically significant difference between the study groups No statistically significant difference between the study groups No statistically significant difference between the study groups

## Data Availability

The data sets used and/or analyzed during the current study are available from the corresponding author on reasonable request.

## Supplementary Materials

Supplementary Materials Supplemental Table 1: search strategy. Supplementary Table 2: GRADE assessment of the evidence.
