# Supplementary material for: Effect of Fentanyl as an Adjuvant to Brachial Plexus Block for Upper Extremity Surgeries: A Systematic Review and Meta-Analysis of RCTs
Source: Pain Res Manag. 2022 Mar 19;2022:8704569. doi: 10.1155/2022/8704569 (PMC8957455; doi:10.1155/2022/8704569)
Supplement: Supplementary Materials — Supplemental Table 1: search strategy. Supplementary Table 2: GRADE assessment of the evidence. [file 8704569.f1.zip › 8704569.f1/Supplementary Table 1.docx]

Supplementary Table 1: Search strategy

| **Query** | **Search Details** |
| --- | --- |
| (fentanyl) AND (axillary block) | ("fentanyl"[MeSH Terms] OR "fentanyl"[All Fields] OR "fentanyls"[All Fields] OR "fentanyl s"[All Fields]) AND (("axilla"[MeSH Terms] OR "axilla"[All Fields] OR "axillary"[All Fields] OR "axillaries"[All Fields] OR "axillaris"[All Fields]) AND ("block"[All Fields] OR "blocked"[All Fields] OR "blocking"[All Fields] OR "blockings"[All Fields] OR "blocks"[All Fields])) |
| (fentanyl) AND (supraclavicular block) | ("fentanyl"[MeSH Terms] OR "fentanyl"[All Fields] OR "fentanyls"[All Fields] OR "fentanyl s"[All Fields]) AND ("supraclavicular"[All Fields] AND ("block"[All Fields] OR "blocked"[All Fields] OR "blocking"[All Fields] OR "blockings"[All Fields] OR "blocks"[All Fields])) |
| (fentanyl) AND (interscalene block) | ("fentanyl"[MeSH Terms] OR "fentanyl"[All Fields] OR "fentanyls"[All Fields] OR "fentanyl s"[All Fields]) AND (("interscalene"[All Fields] OR "interscalenic"[All Fields]) AND ("block"[All Fields] OR "blocked"[All Fields] OR "blocking"[All Fields] OR "blockings"[All Fields] OR "blocks"[All Fields])) |
| (opioids) AND (brachial plexus block) | ("analgesics opioid"[Pharmacological Action] OR "analgesics, opioid"[MeSH Terms] OR ("analgesics"[All Fields] AND "opioid"[All Fields]) OR "opioid analgesics"[All Fields] OR "opioid"[All Fields] OR "opioids"[All Fields] OR "opioid s"[All Fields]) AND ("brachial plexus block"[MeSH Terms] OR ("brachial"[All Fields] AND "plexus"[All Fields] AND "block"[All Fields]) OR "brachial plexus block"[All Fields]) |
| (fentanyl) AND (brachial plexus block) | ("fentanyl"[MeSH Terms] OR "fentanyl"[All Fields] OR "fentanyls"[All Fields] OR "fentanyl s"[All Fields]) AND ("brachial plexus block"[MeSH Terms] OR ("brachial"[All Fields] AND "plexus"[All Fields] AND "block"[All Fields]) OR "brachial plexus block"[All Fields]) |
