# Supplementary material for: Effect of Fentanyl as an Adjuvant to Brachial Plexus Block for Upper Extremity Surgeries: A Systematic Review and Meta-Analysis of RCTs
Source: Pain Res Manag. 2022 Mar 19;2022:8704569. doi: 10.1155/2022/8704569 (PMC8957455; doi:10.1155/2022/8704569)
Supplement: Supplementary Materials — Supplemental Table 1: search strategy. Supplementary Table 2: GRADE assessment of the evidence. [file 8704569.f1.zip › 8704569.f1/Supplementary table 2.docx]

|  | | | | | | | | | | | |
| --- | --- | --- | --- | --- | --- | --- | --- | --- | --- | --- | --- |
| **Certainty assessment** | | | | | | | **Summary of findings** | | | | |
| **Participants  (studies) Follow up** | **Risk of bias** | **Inconsistency** | **Indirectness** | **Imprecision** | **Publication bias** | **Overall certainty of evidence** | **Study event rates (%)** | | **Relative effect (95% CI)** | **Anticipated absolute effects** | |
|  |  |  |  |  |  |  | **With placebo** | **With Fentanyl** |  | **Risk with placebo** | **Risk difference with Fentanyl** |
| **Sensory onset** | | | | | | | | | | | |
| 567 (11 RCTs) | serious ^a^ | not serious | not serious | not serious | none | ⨁⨁⨁◯ MODERATE | 283 | 284 | - | The mean sensory onset was **0** | MD **0.48 lower** (1.81 lower to 0.85 higher) |
| **Motor onset** | | | | | | | | | | | |
| 433 (8 RCTs) | serious ^a^ | not serious | not serious | not serious | none | ⨁⨁⨁◯ MODERATE | 216 | 217 | - | The mean motor onset was **0** | MD **2.36 lower** (3.99 lower to 0.74 lower) |
| **Sensory duration** | | | | | | | | | | | |
| 490 (9 RCTs) | serious ^a^ | not serious | not serious | not serious | publication bias strongly suspected strong association ^b^ | ⨁⨁⨁◯ MODERATE | 245 | 245 | - | The mean sensory duration was **0** | MD **82.81 higher** (41.81 higher to 123.81 higher) |
| **Motor duration** | | | | | | | | | | | |
| 353 (7 RCTs) | serious ^a^ | not serious | not serious | not serious | publication bias strongly suspected strong association ^b^ | ⨁⨁⨁◯ MODERATE | 177 | 176 | - | The mean motor duration was **0** | MD **43.43 higher** (38.98 higher to 47.88 higher) |
| **All complications** | | | | | | | | | | | |
| 385 (4 RCTs) | very serious ^c^ | not serious | not serious | not serious | none | ⨁⨁◯◯ LOW | 16/192 (8.3%) | 32/193 (16.6%) | **OR 2.14** (1.04 to 4.40) | 83 per 1,000 | **80 more per 1,000** (from 3 more to 202 more) |
| **Complications - Nausea vomiting** | | | | | | | | | | | |
| 239 (4 RCTs) | very serious ^c^ | not serious | not serious | not serious | none | ⨁⨁◯◯ LOW | 9/119 (7.6%) | 20/120 (16.7%) | **OR 2.65** (0.73 to 9.53) | 76 per 1,000 | **103 more per 1,000** (from 19 fewer to 362 more) |
| **Complications - Pruritis** | | | | | | | | | | | |
| 146 (3 RCTs) | serious ^d^ | not serious | not serious | not serious | none | ⨁⨁⨁◯ MODERATE | 7/73 (9.6%) | 12/73 (16.4%) | **OR 1.86** (0.66 to 5.23) | 96 per 1,000 | **69 more per 1,000** (from 30 fewer to 261 more) |

**CI:** Confidence interval; **MD:** Mean difference; **OR:** Odds ratio

#### Explanations

a. Overall risk of bias noted to have "some concerns" in multiple studies

b. Evidence of publication bias

c. Two of the four studies had "high risk" of bias

d. One study had "high risk" of bias
